# Thiocillin contributes to the ecological fitness of *Bacillus cereus* ATCC 14579 during interspecies interactions with *Myxococcus xanthus*

**DOI:** 10.3389/fmicb.2023.1295262

**Published:** 2023-11-24

**Authors:** Susanne Müller, Orlando DeLeon, Samantha N. Atkinson, Fatima Saravia, Stephanie Kellogg, Elizabeth A. Shank, John R. Kirby

**Affiliations:** ^1^Department of Microbiology & Immunology, Medical College of Wisconsin, Milwaukee, WI, United States; ^2^Department of Medicine, University of Chicago Pritzker School of Medicine, Chicago, IL, United States; ^3^Department of Systems Biology, University of Massachusetts Chan Medical School, Worchester, MA, United States

**Keywords:** predator–prey interactions, competition, specialized metabolites, *M. xanthus*, *B. cereus*

## Abstract

The soil-dwelling delta-proteobacterium *Myxococcus xanthus* is a model organism to study predation and competition. *M. xanthus* preys on a broad range of bacteria mediated by lytic enzymes, exopolysaccharides, Type-IV pilus-based motility, and specialized metabolites. Competition between *M. xanthus* and prey bacterial strains with various specialized metabolite profiles indicates a range of fitness, suggesting that specialized metabolites contribute to prey survival. To expand our understanding of how specialized metabolites affect predator–prey dynamics, we assessed interspecies interactions between *M. xanthus* and two strains of *Bacillus cereus*. While strain ATCC 14579 resisted predation, strain T was found to be highly sensitive to *M. xanthus* predation. The interaction between *B. cereus* ATCC 14579 and *M. xanthus* appears to be competitive, resulting in population loss for both predator and prey. Genome analysis revealed that ATCC 14579 belongs to a clade that possesses the biosynthetic gene cluster for production of thiocillins, whereas *B. cereus* strain T lacks those genes. Further, purified thiocillin protects *B. cereus* strains unable to produce this specialized metabolite, strengthening the finding that thiocillin protects against predation and contributes to the ecological fitness of *B. cereus* ATCC 14579. Lastly, strains that produce thiocillin appear to confer some level of protection to their own antibiotic by encoding an additional copy of the L11 ribosomal protein, a known target for thiopeptides. This work highlights the importance of specialized metabolites affecting predator–prey dynamics in soil microenvironments.

## 1 Introduction

Predation is a common form of interspecies interaction in ecosystems. Bacterial predators are important drivers shaping ecological communities: they affect microbial diversity and composition and act as an evolutionary force in bacterial selection (Johnke et al., 2014; Koskella and Brockhurst, 2014; Karakoc et al., 2017; Nair et al., 2019; Wang et al., 2019). Predatory myxobacteria like *Myxococcus xanthus* secrete hydrolytic enzymes that degrade prey into consumable molecules (Hart and Zahler, 1966; Sudo and Dworkin, 1972; Munoz-Dorado et al., 2016). *M. xanthus* has a broad prey spectrum ranging from bacteria to yeast and phage, which makes it a major contributor shaping microbial populations in the environment (Morgan et al., 2010; Muller et al., 2014; Livingstone et al., 2018). *M. xanthus* also produces specialized metabolites, two of which have been shown to affect predation (Findlay, 2016; Bader et al., 2020): myxovirescin targets signal peptidase LspA from *Escherichia coli* to enhance predation (Xiao et al., 2012), and myxoprincomide provides protection for *M. xanthus* against *Bacillus subtilis* to promote predation (Muller et al., 2016).

Specialized metabolites are also important for defense and multiple strategies for avoidance have been identified. For example, *Streptomyces coelicolor* produces actinorhodin in response to *M. xanthus* predation, while *Bacillus licheniformis* escapes predation by deactivating myxovirescin (Perez et al., 2011; Wang et al., 2019). Additionally, we have shown that *B. subtilis* NCIB3610 produces bacillaene to delay predation by *M. xanthus* until *B. subtilis* is capable of sporulating within megastructures (Muller et al., 2014). Thus, evidence continues to accumulate that specialized metabolites are part of both predatory and survival mechanisms.

To expand our understanding of *M. xanthus* predator–prey interactions, we conducted a screen to identify susceptible or resistant bacterial strains commonly found in soil. In a companion study (unpublished data), we assayed 5 predatory Myxococcales strains (*M. xanthus*, *Stigmatella aurantiaca, M. flavescens*, *M. virescens*, and *M. fulvus*) for their ability to induce megastructure formation (Muller et al., 2015) in 9 related Bacillales strains (*B. subtilis* 3610, *B. cereus* strains T and ATCC 14579, *B. thuringiensis*, *B. licheniformis*, *B. pumilis*, *Paenibacillus alvei*, and *P. polymyxa* strains ATCC 842 and KCT 3627). From that initial screen, two *B. cereus* stains displayed differential susceptibility to predation: strain ATCC 14579 is resistant to predation whereas strain T is sensitive. We therefore hypothesized that strain ATCC 14579 may produce a specialized metabolite responsible for resistance to predation by *M. xanthus*.

Genome sequencing and analysis revealed that strain T lacks the core components of the biosynthetic gene cluster (BGC) for thiocillins, whereas the predation-resistant 14579 encodes all components for thiocillin biosynthesis. Thiocillins are ribosomally encoded, post-translationally modified, thiazolyl peptide antibiotics and are known to inhibit the interaction between the 23S rRNA and protein L11 within the 50S ribosomal subunit of Gram-positive bacteria (Cameron et al., 2002; Harms et al., 2008). Thiocillin precursors undergo extensive posttranslational modifications on the C-terminus of the pre-peptide, generating a set of eight related antibiotics (Acker et al., 2009; Wieland Brown et al., 2009). Additionally, we performed a phylogenetic analysis of 109 *B. cereus* strains, which revealed that 14579 belongs to a clade possessing the thiocillin BGC while other close relatives do not. Thus, it appears that the thiocillin BGC was horizontally acquired from other bacteria, consistent with previous findings (Grubbs et al., 2017). Furthermore, each thiocillin BGC encodes at least one paralog for L11, providing a potential mechanism for resistance for the producing strains, as previously suggested (Acker et al., 2009; Wieland Brown et al., 2009; Bowers et al., 2010).

Our predation assays here demonstrate that 14579 mutants lacking thiocillin precursor-encoding genes are sensitive to *M. xanthus* predation, while the addition of purified thiocillin rescues these same mutants. Lastly, we observed that viability of *M. xanthus* was strongly affected during competition with 14579 cells in a thiocillin-dependent manner, strongly suggesting that the antimicrobial activity of thiocillins goes beyond Gram-positive bacteria, although the mechanism of action has not been determined. To our knowledge, thiocillin is the first specialized metabolite known to directly affect the fitness of *M. xanthus*. Overall, these findings show that thiocillin provides an advantage for *B. cereus* during competition with a naturally co-occurring predator, *M. xanthus*.

## 2 Materials and methods

### 2.1 Bacterial strains and media

*Myxococcus xanthus* and *Bacillus* strains used in this study are listed in Table 1. *M. xanthus* cultures were grown in liquid CYE media to mid-log phase before processing (Bretscher and Kaiser, 1978). *Bacillus* strains were grown in liquid LB media to a final optical density of about OD_600nm_ of 2.

**Table 1:** Bacterial strains used in this study.

Bacterial strains	Reference/Source
*Myxococcus xanthus* DZ2	Muller et al. (2014)
DZ2 *attB8*::pWB200	Muller et al. (2016)
*Bacillus cereus* strain T	Bacillus genetic stock center
*Bacillus cereus* ATCC14579	Bacillus genetic stock center
*Bacillus cereus* ATCC14579 ***∆**tclE-H*	Bleich et al. (2015)
*Bacillus cereus* ATCC14579 *tclE-H*::*tclE* (T4V)	Bleich et al. (2015)
*Bacillus cereus* ATCC14579 ***∆**tclM*	Bleich et al. (2015)
*Bacillus cereus* ATCC14579 *tclE-H*::*tclE* (A78)	Bleich et al. (2015)
*Bacillus cereus* ATCC14579 *tclE-H*::*tclE* (C9A)	Bleich et al. (2015)
*Bacillus cereus* ATCC14579 *tclE-H*::*tclE* (C5A)	Bleich et al. (2015)
*Bacillus cereus* ATCC14579 *tclE-H*::*tclE* (C7A)	Bleich et al. (2015)
*Bacillus cereus* ATCC14579 *tclE-H*::*tclE* (C2S)	Bleich et al. (2015)

### 2.2 Interspecies interaction assays

The *M. xanthus* predator strain DZ2 was washed twice with MMC buffer to remove all nutrients and diluted in MMC buffer (10 mM MOPS [morpholinepropanesulfonic acid; pH 7.6], 4 mM MgSO_4_, 2 mM CaCl_2_) to a final concentration of 2×10^9^ cells/ml (Muller et al., 2014). Predator and prey cells (*B. cereus* strains at a final concentration of 1×10^11^ cells/ml) were mixed at a ratio of 1:50 and 7 μL were spotted onto CFL starvation media [10 mL 1 M MOPS pH 7.6, 10 mL 0.1 M KH_2_PO_4_, 10 mL 0.8 M MgSO_4_, 10 mL 2% (NH_4_)_2_SO_4_, 1 mL 20% Sodium Citrate, 0.015 g Casitone, 15 g agar, 959 mL H2O] (Berleman and Kirby, 2007) and incubated at 32°C. As a negative control, MMC buffer alone was used instead of the predator. Predation is displayed in form of lysis of the prey cells compared to the control. To test whether purified thiocillin affects predation, interspecies interaction assays were set up as described above and thiocillin or thiocillin T4V (dissolved in DMSO) were added at a final concentration of 330–990 ng/microliter. As a negative control, DMSO alone, at the same concentration was used instead of the predator. Native thiocillin was purified or obtained from Cayman Chemical or from Albert Bowers (UNC-Chapel Hill, provided to E.A.S.) (Acker et al., 2009; Bowers et al., 2012). Molecules from each source yielded similar results. Purified thiocillin T4V is not commercially available and was obtained from Albert Bowers.

### 2.3 Microscopy

All assays were monitored by microscopy using a Nikon SMZ10000 dissecting microscope. Images were taken using a QImaging Micropublisher CCD camera and processed with Qcapture software.

### 2.4 PacBio sequencing and assembly

*Bacillus cereus* strains T and ATCC 14579 were sequenced at the University of Wisconsin Milwaukee School Freshwater Sciences Great Lakes Genomic Center using the Pacific Biosciences (PacBio) RSII SMRTcell system. Resulting reads were then assembled using Canu v.1.6 (Koren et al., 2017) with default settings except for a corrected error rate of 0.040 and the respective genome size of 5.76 MB for *B. cereus* strains. Circlator v. 1.5.5 (Hunt et al., 2015) was used to circularize the genomes and verified with MUMmer v. 3.23 (Kurtz et al., 2004) to confirm the correct regions were matched. Circularized genomes were then annotated on the RAST server^1^ (Aziz et al., 2008; Brettin et al., 2015). The nucleotide sequence for *B. cereus* strain T has been submitted to GenBank under nucleotide accession number CP130491. The nucleotide sequence for *B. cereus* ATCC 14579 is available under nucleotide accession number CP138336, CO138337.

### 2.5 Pangenomics analysis

Pangenomics analysis of the *B. cereus* strains were performed using the anvio suite (v5.5) (Eren et al., 2015; Delmont and Eren, 2018). FASTA files for the *B. cereus* strains T and ATCC 14579 were annotated for open reading frames (ORFs) using Prodigal under default settings. Subsequent ORF calls were translated and given protein annotations using the PFAMs database (Jones et al., 2014) under “sensitive” settings. Biosynthetic gene clusters (BGCs) were identified using antiSMASHv5 under default settings (Blin et al., 2019). FASTA files were then converted into anvio contigs databases (“anvi-gen-contigs-database”) with the ORF calls, PFAM calls, and BGC annotations imported using the “anvi-import”. A custom python script was used to parse the antiSMASH outputs to the anvio import format. Single copy genes in each genome were identified using a hidden markov model (hmm) with the Campbell database for reference (“anvi-run-hmms”) (Campbell et al., 2013). To perform the pangenomics analysis, a single genomes database file was generated to integrate the two contigs database files for the two strains (“anvi-gen-genomes-storage” using the flag “—external-genomes”). The pangenome analysis was performed under the command “anvi-pan-genome” using the settings “—minbit = 0.5”, “—mcl-inflation = 10”, and “—use-ncbi-blast”. Briefly, NCBI BLASTP was used to generate matches between the translated ORFs of the two strains. The subsequent protein cluster matches were then refined using the minbit heuristic as defined by Benedict et al. (2014). Cluster granularity and sensitivity was further refined by the MCL inflation parameter =10 (high sensitivity, low granularity) as T and ATCC 14579 are strains of the same species and closely related. The resulting protein clusters were then visualized using “anvi-display-pan” and sorted using a “presence or absence” scheme including protein clusters belonging to the thiocillin cluster and other BGCs flagged. Further visual manipulations were performed in Inkscape.

### 2.6 Phylogenomics analysis

To provide phylogenetic relationships between the *B. cereus* species relative to the presence of the thiocillin BGC, we examined 109 publicly available strains’ genomes (from the RefSeq database) including the original ATCC 14579 sequence (accession number AE016877.1). See Supplementary Table S1 for strain names. FASTA files for these genomes, along with those for strain T and ATCC 14579 were annotated and imported into anvio for a pangenomics analysis as described above. All open reading frames were reannotated using Prodigal for consistency and anvio formatting purposes. For phylogenomic comparisons, single-copy genes found to be shared by the 111 strains were called using “anvi-get-sequences-for-hmm-hits” using the flags “--hmm-source Campbell_et_al”, “--get-aa-sequences”, and “—concatenate” (Campbell et al., 2013). The resulting FASTA file output had a resulting aa sequence of the concatenated single-copy genes for each genome. Sequences were then aligned using MUSCLE (Edgar, 2004) and a tree was generated using an approximately maximum-likelihood method (ML) and exported into Newick (.nwk) format (Edgar, 2004; Price et al., 2010). The resulting tree was midpoint rooted and visualized in FigTree (Rambaut, A., Drummond AJ 2010 FigTree v1.3.1. Institute of Evolutionary Biology, University of Edinburgh, Edinburgh http://tree.bio.ed.ac.uk/software/figtree/).

## 3 Results

### 3.1 Production of thiocillin protects *Bacillus cereus* ATCC 14579 from predation by *Myxococcus xanthus*

**Figure 1:**
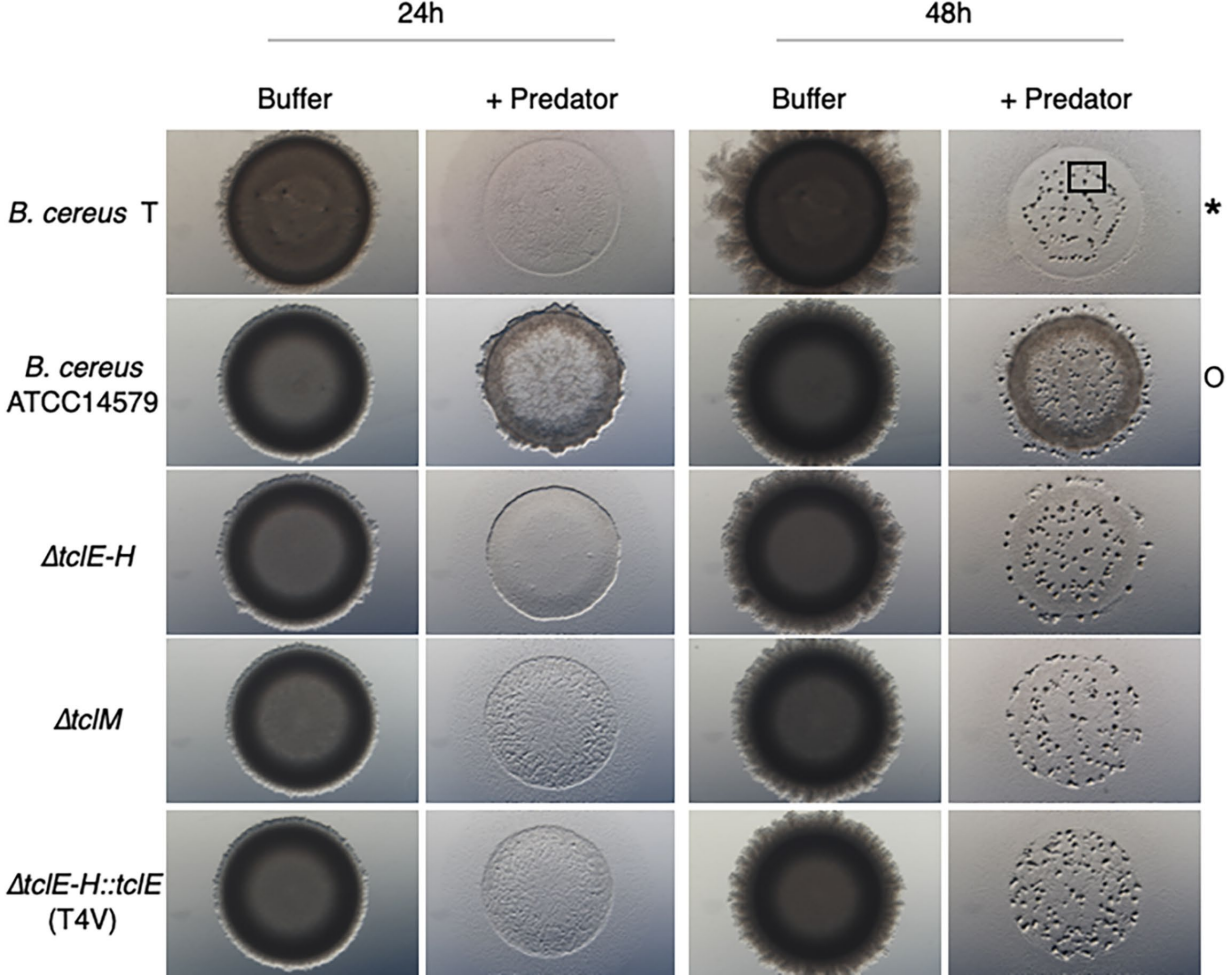
Thiocillin protects *B. cereus* ATCC 14579 from predation by *M. xanthus*. *B. cereus* strains (prey) were grown to a certain cell density (see Materials and Methods) and nutrients were removed by washing the cells multiple times. Prey cells were mixed with the predator *M. xanthus* in a ratio of 50:1, spotted on CFL Agar plates and incubated at 32°C. Pictures were taken after different times of incubation at 15x magnification. Predation is visible by cell lysis (*) whereas competition/predation resistance is indicated by minimal loss of cells (O). Fruiting body formation can be seen after 48 h as an indicator that predator *M. xanthus* sensed a drop down in nutrients and is starving (black square). Only *B. cereus* ATCC 14579 resists predation and mutations inhibiting thiocillin production or resulting in modifications of the thiocillin molecule render cells to be predation sensitive. *B. cereus* ATCC 14579 *∆tclE-H* does not contain the thiocillin prepeptide encoding genes *tclE-H* and does therefore not make thiocillin. *B. cereus* ATCC 14579 *∆tclE-H::tclE* (T4V) makes a variant of the thiocillin molecule that has lost the antimicrobial function of the molecule but is still able to induce matrix formation in *B. subtilis*. Strain *∆tclM* produces a thiocillin-like molecule that has no closed ring structure.

While *M. xanthus* can prey upon a large variety of bacteria, some strains are resistant to predation (Muller et al., 2014). We conducted a screen that revealed that *B. cereus* strain T is sensitive to *M. xanthus* predation while *B. cereus* ATCC 14579 is resistant. Both predator and prey cells were grown as described in Materials and Methods, washed, mixed and then spotted onto agar plates (See Methods). Predation is visible as clearing of the prey colony relative to the buffer control, while resistance is indicated by a ring of cells at the edge of the original spot (Figure 1). Colonies form a doughnut shape with higher cell density at the outer edge of the spot due to surface tension. After suitable prey is consumed, *M. xanthus* cells undergo fruiting body formation as described previously (Berleman and Kirby, 2007) and is visible as dark aggregates after 48 h (square in Figure 1). The results indicate that *B. cereus* strain T is consumed while strain 14579 is largely resistant to predation by *M. xanthus* under the conditions of our assay. In addition, few *M. xanthus* cells appear outside of the original spot of 14579 (Supplementary Figure S1), suggesting that predation or motility may be reduced for *M. xanthus* cells due to an inhibitory factor produced by 14579.

Because specialized metabolites contribute to interspecies interactions, and due to thiocillin’s established role as both a killing and signaling molecule, we hypothesized that *B. cereus* ATCC 14579 is resistant to predation by *M. xanthus* due to thiocillin production (Xiao et al., 2012; Muller et al., 2014; Bleich et al., 2015; Muller et al., 2015; Findlay, 2016; Muller et al., 2016). Therefore, we tested various 14579 thiocillin mutant strains for their ability to resist or compete with *M. xanthus*. 14579 mutant cells lacking genes encoding the thiocillin precursor peptide (*∆tclE-H*) were found to be sensitive to predation by *M. xanthus*, like strain T. Furthermore, we tested various mutant thiocillin strains (T4V, A78, C9A, C5A, C7A, and C2S) each of which lack antimicrobial activity against *B. subtilis* but retain the capacity to induce biofilm formation in *B. subtilis* (Acker et al., 2009; Bleich et al., 2015). We also tested a *∆tclM* mutant strain that produces a thiocillin derivative with no macrocycle ring. Each *B. cereus* thiocillin mutant strain was sensitive to predation by *M. xanthus* (Figure 1 and Supplementary Figure S2). We conclude that the *B. cereus* wild-type thiocillin confers resistance to *M. xanthus* predation.

### 3.2 Purified thiocillin protects sensitive *Bacillus cereus* strains from predation by *Myxococcus xanthus*

**Figure 2:**
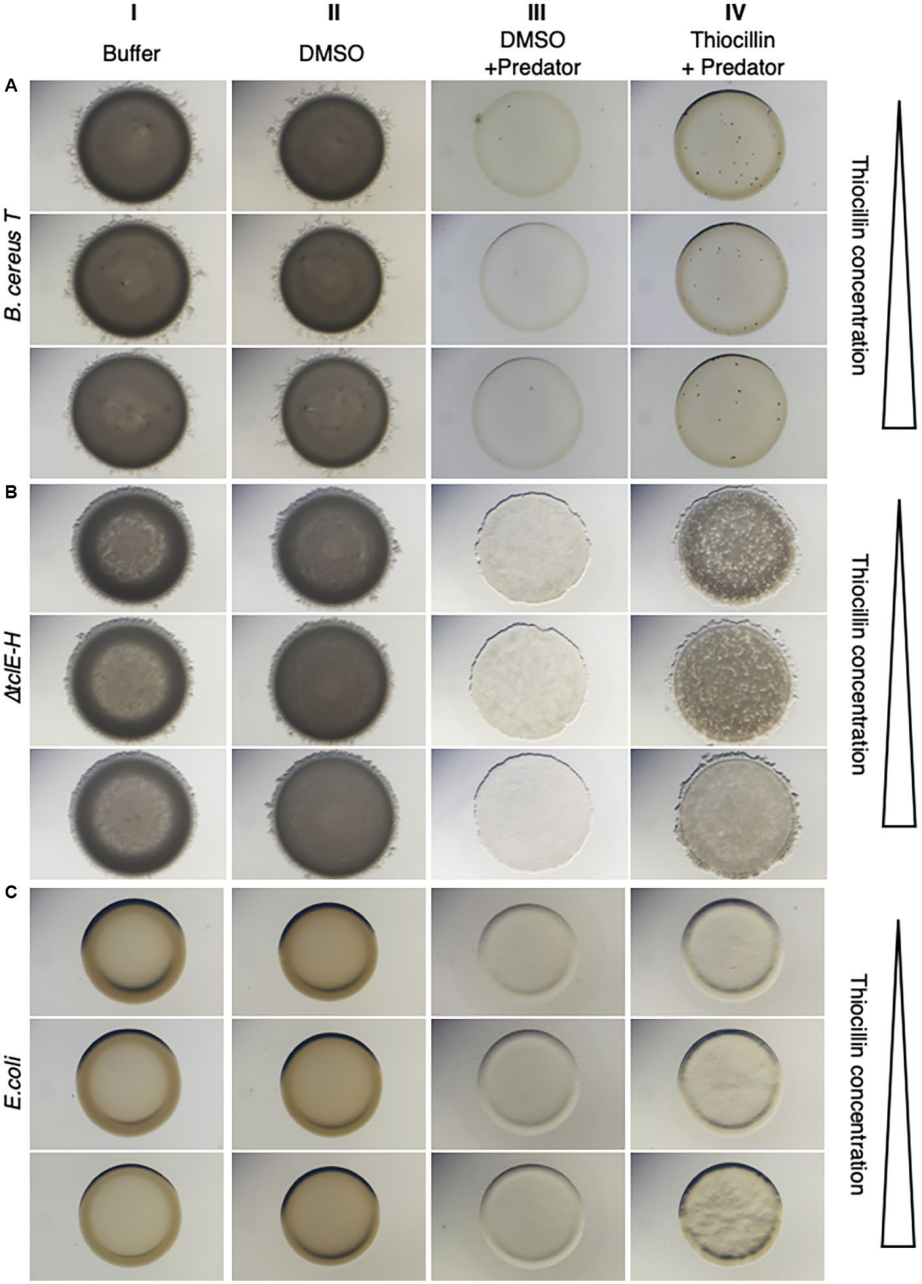
Purified Thiocillin rescues sensitive strains from predation by *M. xanthus*. The predation sensitive strains *B. cereus* strain T **(A)**, *B. cereus* ATCC 14579 *∆tclE-H* **(B)** and *E.coli DH5α* **(C)** were tested in predation assays with the predator *M. xanthus* with and without purified thiocillin (dissolved in DMSO). *B. cereus* ATCC 14579 *∆tclE-H* is not able to produce thiocillin anymore and *B. cereus* strain T does not contain the thiocillin BSG. All strains are sensitive to predation (comparing column III to I and II). The addition of purified thiocillin protected all sensitive strains from predation (column IV). Increasing concentrations of thiocillin enhanced the predation protective effect. Pictures were taken after 24 h.

To further assess the ability for thiocillin to protect against predation, we tested purified thiocillin for its ability to rescue predation-sensitive mutants. We also tested the purified T4V variant of thiocillin, since this molecule was shown to have no antimicrobial activity against *B. subtilis* or *Staphylococcus aureus* (Acker et al., 2009). The purified molecules were dissolved in DMSO to a final concentration up to 1 μg/μl. Assays were performed by mixing *M. xanthus* and *B. cereus* cells with and without purified thiocillin or vehicle alone (see Methods, Figure 2). We tested both *B. cereus* strain T (Figure 2A) and 14579 *∆tclE-H* mutant cells (Figure 2B). *B. cereus* cells alone show no clearing (Figure 2, columns I and II). When *M. xanthus* is added, the prey cells are consumed resulting in clearing (Figure 2, column III). In contrast, purified thiocillin appears to increase survival of *B. cereus*, which was more pronounced with increasing concentrations of thiocillin (Figure 2, column IV). Protection from predation was also visible using *E. coli* and the *B. cereus* 14579 T4V mutant cells supplied with extracellular thiocillin (Figure 2C and Supplementary Figure S3). We also observed *M. xanthus* cells beyond the edge of the original prey spot when thiocillin was present at low concentrations but not at high concentrations, further suggesting an inhibitory role for thiocillin against *M. xanthus* (Supplementary Figure S4). We conducted a parallel set of assays using purified T4V (Supplementary Figure S5). The T4V variant of thiocillin was unable to protect sensitive cells against predation by *M. xanthus*. Together, these results show that exogenous thiocillin can protect sensitive *B. cereus* strains, as well as *E. coli*, from *M. xanthus* predation.

### 3.3 Thiocillin affects the fitness of *Myxococcus xanthus* in predation assays with *Bacillus cereus* 14579

**Figure 3:**
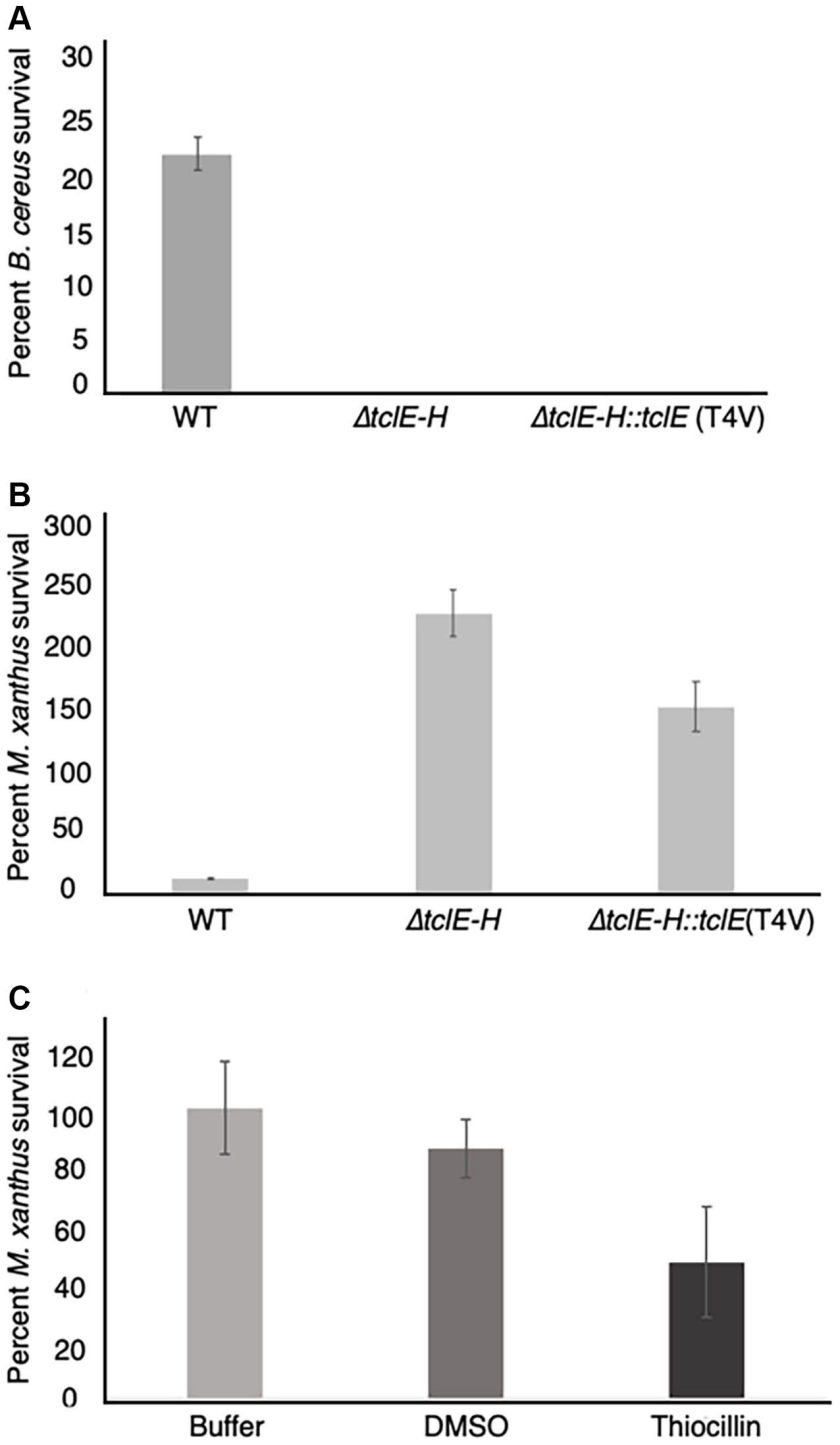
Quantification of predator and prey survival reveals strong competition during interspecies interactions between *M. xanthus* and *B. cereus* ATCC 14579. Quantification of prey survival and predator survival/growth. **(A)** Prey *B. cereus* ATCC 14579 and mutant strains *B. cereus* ATCC 14579 *∆tclE-H* or *∆tclE-H::tclE* (T4V) were mix in a 50:1 ratio with *M. xanthus* and spotted onto CFL Agar plates. As controls the strains were incubated individually. After incubation at 32°C. for 24 h, cells were removed from the plates and CFU were determined to calculate prey survival calculated relative to controls. The majority of *B. cereus* ATCC 14579 *∆tclE-H* and *∆tclE-H::tclE* (T4V) were consumed by the predator *M. xanthus*, whereas about 23.5% of the wild type strain *B. cereus* ATCC 14579 survived. **(B)** *M. xanthus* was able to grow in the presence of *B. cereus* ATCC 14579 *∆tclE-H* and *∆tclE-H::tclE* (T4V) but only around 12% of *M. xanthus* could be recovered when mixed with *B. cereus* ATCC 14579 clearly showing this interspecies interaction is of competitive nature effecting the ecological fitness of both strains. **(C)** Purified thiocillin affects predator viability. Percent *M. xanthus* survival after 24 h. *M. xanthus* cells where washed and resuspended to 250 KU. Cells where mixed with either buffer, DMSO or thiocillin (thiocillin 300 ng) and spotted on CFL Agar. Cells were harvested after 24 h and serial dilutions were plates in CYE agar to calculated CFU’s.

The above experiments (Figure 1) reveal that thiocillin confers resistance to predation by *M. xanthus* and suggest a potential to inhibit predator cells. To test this possibility, we conducted additional quantitative experiments to assess survival rates for both *M. xanthus* and *B. cereus* in our assays. To conduct these experiments, both wild-type and mutant *B. cereus* strains were mixed with *M. xanthus* cells and incubated as described above. For controls, and to normalize the results, each strain was also incubated alone on agar surfaces. Cells were harvested after 24 h and colony forming units (CFU) were determined on selective media that supported either the growth of *B. cereus* (LB) or *M. xanthus* (CYE with kanamycin). *B. cereus* strains were evaluated at 24 h when no growth of *M. xanthus* was detected. The results were normalized to the starting number of CFUs for each experiment (Figure 3, Methods). In agreement with the visual phenotypic data (Figure 1), *B. cereus* mutants (*∆tclE-H* and T4V-producing strains) showed survival rates that were below 1%. In contrast, about 23% of the *B. cereus* ATCC 14579 cells survived, indicating that thiocillin provides a distinct advantage against predation by *M. xanthus* (Figure 3A).

Predators typically grow because of the nutrient uptake from killing and consuming prey. This was observed for *M. xanthus*, which displayed growth of ~230% when mixed with *B. cereus* strain *∆tclE-H* and ~ 150% when mixed with *B. cereus* T4V compared to growth alone. In contrast, only about 12% of *M. xanthus* cells survived the interaction with *B. cereus* 14579 (Figure 3B). Together, these results indicate that production of native thiocillin not only enhances *B. cereus* survival in the presence of *M. xanthus* but also that thiocillin reduces *M. xanthus* survival under the conditions of our assay. We next tested the effect of purified thiocillin directly on *M. xanthus* cells and plated as above to determine CFU. The addition of thiocillin resulted in a ~ 50% decrease in survival of *M. xanthus* when compared to controls (Figure 3C). These results suggest that thiocillin exerts its protective effect by reducing the viability of the predator.

### 3.4 Comparative genomics of *Bacillus cereus* strain T and strain ATCC 14579 reveals a difference in the biosynthetic gene cluster responsible for thiocillin production

**Figure 4:**
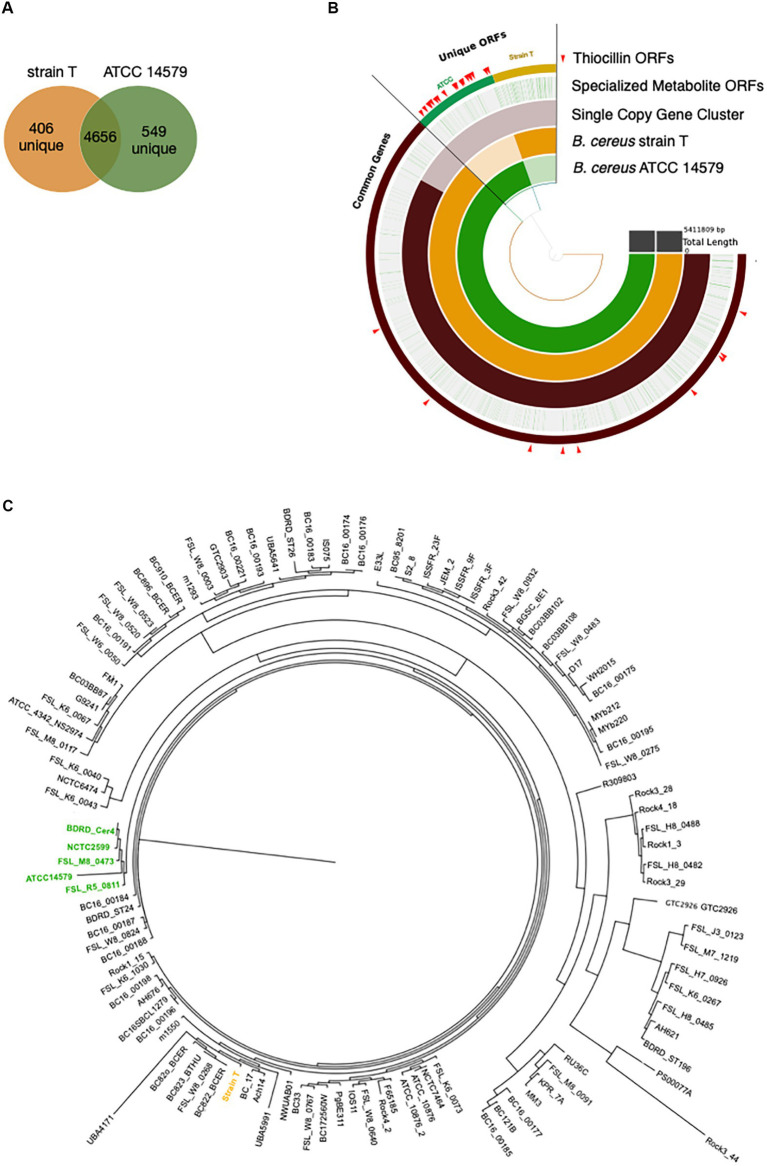
Pangenomics of *B. cereus* strain T and ATCC 14579 reveals differences in specialized metabolite gene clusters. **(A)** Venn diagram of the shared genes and unique genes between *B. cereus* strain T and ATCC 14579. **(B)** Anvi’o plot of *B. cereus* strain T and ATCC 14579 genomes. Open reading frames (ORFs) for the two strains were compared using the anvio workflow for pangenomics. ORFs were called using prodigal and annotated using InterproScan for PFAM annotations. ORFs were aligned and compared using blastp and weak matches refined using minbit = 0.5 and MCL inflation parameter of 10. AntiSMASHv5.0 was used to annotate the complete specialized metabolite clusters, including thiocillin. Individual ORFs for all specialized metabolites are shown in green lines (Specialized Metabolite ORFs ring), many of which are specific to ATCC (green) and strain T (gold) as visible in the thiocillin ORFs ring. ORFs comprising the thiocillin operon are marked with red arrows. **(C)** Phylogenetic tree (Maximum Likelihood, midpoint root) based on a multiple sequence alignment of SCGs (single copy genes) using 109 *B. cereus* strains. Five thiocillin producing strains were identified that cluster together (green) indicating that they are closely related. Due to the close phylogenetic relationship of the thiocillin BGC containing strains it is most likely that a single common ancestor did gain the BGC by horizontal gene transfer.

The above data demonstrate a role for thiocillin in protecting *B. cereus* from *M. xanthus* predation. Furthermore, the phenotypic differences between strain T and 14579 suggest that thiocillin production likely does not occur for strain T. We predicted that these strains would harbor changes in biosynthetic genes required for production or delivery of thiocillin. Thus, we utilized PacBio long-read sequencing to determine genomic content for each strain. The Anvi’o workflow for pangenomics was used to compare both genomes (Figure 4B). Overall, we found 4,656 shared gene clusters between the two strains, 406 gene clusters exclusive to strain T, and 549 gene clusters exclusive to 14579 (Figure 4A). The term “gene cluster” is defined as sequences of one or more predicted open reading frames grouped together based on homology from translated DNA sequences. Gene clusters may contain orthologous or paralogous sequences from one or more genomes analyzed within the pangenome (Delmont and Eren, 2018).

**Figure 5:**
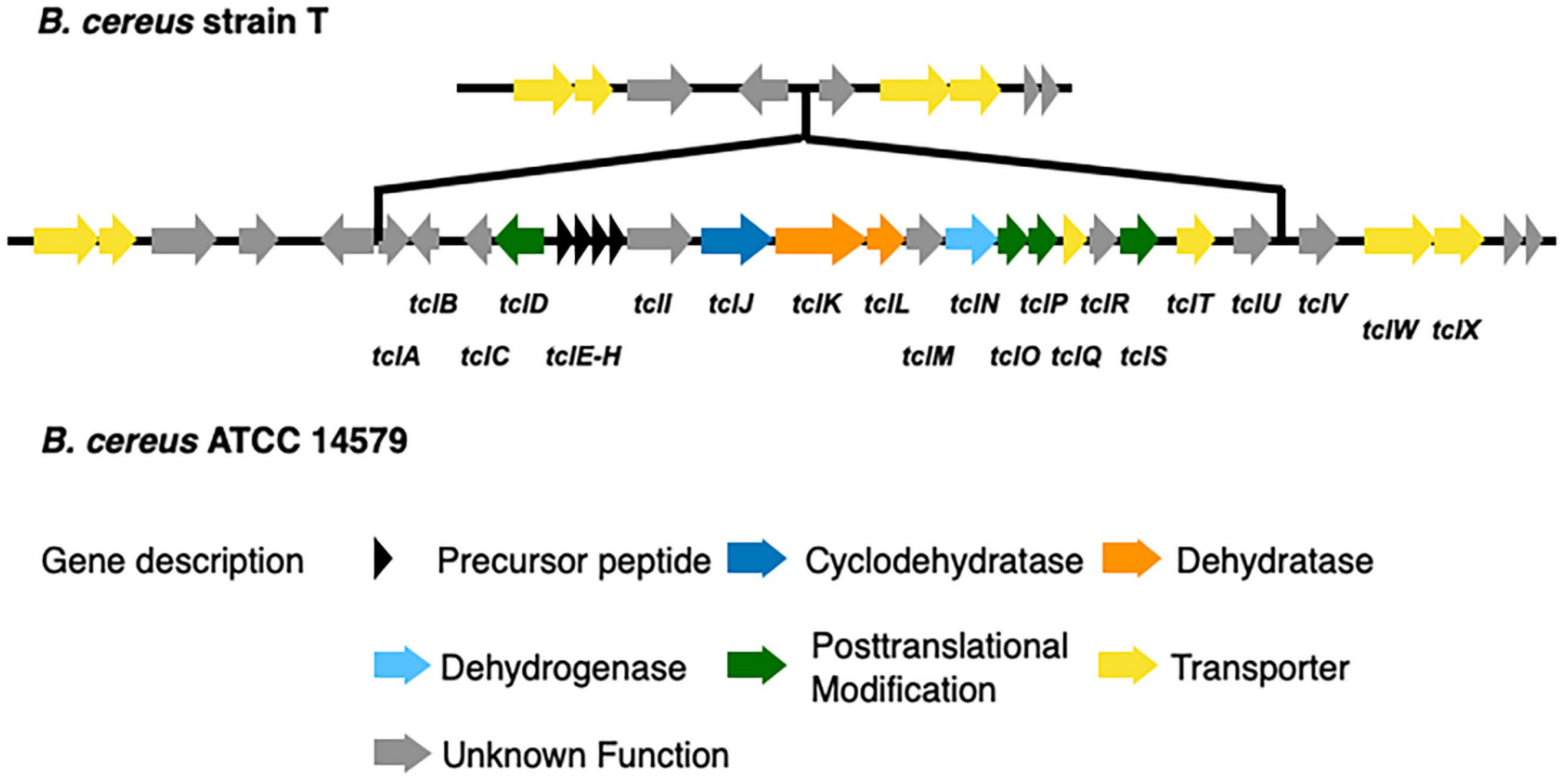
Comparison of the thiocillin synthesis gene clusters of *B. cereus* ATCC 14579 and *B. cereus* T. The thiocillin synthesis gene cluster is composed of the precursor peptide encoding genes *tclE-H* (black) as well as biosynthesis genes (blue and orange). Genes encoding proteins functioning in posttranslational modification (green) and transport (yellow) are also part of the thiocillin synthesis gene cluster. Comparison of both *B. cereus* strains revealed that *B. cereus* T does not contain the central components of the thiocillin synthesis gene cluster t*clA-tclU* including the precursor peptide encoding genes *tclE-H*.

We identified 9 BGCs common to both genomes including 3 non-ribosomal peptide synthases as well as other BGCs required for synthesis of 3 bacteriocins, 1 betalactone, 1 siderophore, and 1 terpene. Strain T has 3 additional BGCs not found in 14579 (Table 2) while 14579 has 1 BGC predicted to generate thiocillin, which was not found in strain T (Blin et al., 2019). The thiocillin BGC in 14579 includes the thiocillin-precursor encoding genes *tclE-H* as well as core genes involved in biosynthesis, posttranslational modification, transport, and additional genes (Figure 5). Notably, strain T lacks open reading frames encoding TclA-TclU, which include most of the core functions for production of thiocillin. However, strain T does possess transporter functions common to strain 14579, suggesting either an insertion or deletion of the core biosynthetic genes for thiocillin occurred in a common ancestor. Overall, *B. cereus* strain T appears to be incapable of thiocillin production.

**Table 2:** Comparison of BGCs found in *B. cereus* strain T and ATCC14579.

Type	% Nucleotide similarity of core BGCs ORFs
Similar BGCs	
Bacteriocin	98.13%
Bacteriocin	97.88%
Bacteriocin	91.41%
Betalactone	97.51%
NRPS	98.40%
NRPS	98.31%
NRPS	92.59%
Siderophore	98.42%
Terpene	98.84%
Unique BGCs	
Thiopeptide (thiocillin)	ATCC14579
NRPS/transAT-PKS	strain T
NRPS	strain T
NRPS	strain T

Furthermore, the BGC in 14579 encodes two copies of a paralog of the L11 ribosomal protein encoded by *rplK* located near genes encoding the alpha and beta subunits of RNA polymerase, *rpoA* and *rpoB*, respectively. L11 associates with the 23S rRNA within the 50S subunit of bacterial ribosomes and is the known target for thiocillins (Cameron et al., 2002; Harms et al., 2008). The two paralogs of L11 encoded within the thiocillin BGC for 14579, TclQ and TclT, are identical to each other but distinct from L11 (Supplementary Figure S6). As previously described, the L11 paralogs likely provide protection for thiocillin-producing strains, as suggested previously (Acker et al., 2009; Wieland Brown et al., 2009; Bowers et al., 2010). Whether or not TclQ and TclT can interact directly or indirectly with 23S rRNA or within the 50S subunit has not been determined experimentally.

To address the question of whether the core BGC differences between 14579 and strain T likely resulted from insertion or deletion, we analyzed 109 publicly available *B. cereus* genomes for the presence or absence of 21 core thiocillin biosynthetic genes within the group (Figure 4C). We generated a multiple sequence alignment of all shared proteins between these strains using MUSCLE and examined their phylogenetic relationships using a maximum likelihood tree (Edgar, 2004). We then examined the genomes for each gene within the known thiocillin BGC spanning *tclA-tclX* and utilized antiSMASH to assess each genome for the presence of a thiocillin-encoding BGC. Of the 109 strains, only five contained the biosynthetic gene cluster for thiocillin (strains FSL_R5_0811, FSL_M8_0473, NCTC2599, BDRD_Cer4, and ATCC 14579), consistent with previous reports indicating that only 4% of *B. cereus* strains possess thiocillin-like BGCs (Grubbs et al., 2017). Each of these 5 strains encode at least one paralog of the L11 ribosomal protein. Other than the 5 strains listed above, the remaining 104 *B. cereus* genomes lack the core *tclA-tclX* genes based on a BLAST-P cutoff (E < 10e-5) utilized by antiSMASH (Blin et al., 2019). Overall, the close phylogenetic relationship between the thiocillin-BGC-containing *B. cereus* strains identified here indicate that a single common ancestor likely acquired the thiocillin BGC as a gain of function for the clade represented in Figure 4C.

## 4 Discussion

Competition between different bacterial species is diverse and relies on many different strategies. In this study, we have focused on the use of specialized metabolites to influence predator–prey dynamics thought to occur in soil microenvironments. We show that *Bacillus cereus* protects itself from predation by *Myxococcus xanthus* and that the specialized metabolite, thiocillin, is responsible for the observed protection. We performed assays using a thiocillin mutant strain (∆*tclE-H*) as well as several strains that produce variant forms of thiocillin (e.g., T4V); the assays reveal that only native thiocillin promotes survival of *B. cereus* in the presence of *M. xanthus*. We also show that purified thiocillin transiently protects non-thiocillin producing strains in a dose-dependent manner, including *E. coli*, from predation by *M. xanthus*. Together, these results indicate that the specialized metabolite, thiocillin, is important for protection from *M. xanthus* predation.

Thiopeptides have not been widely reported to have antimicrobial activity against proteobacteria, although *Pseudomonas aeruginosa* and *Acinetobacter baumannii* were found to be sensitive to thiostrepton (Ranieri et al., 2019), and *P. aeruginosa* is sensitive to thiocillin and micrococcin (Chan and Burrows, 2021). Here we show that thiocillin produced by *B. cereus* ATCC 14579 significantly affects *M. xanthus* survival during predation assays, and that both predator and prey display losses in viability when thiocillin is present. In contrast, the antibiotic-null T4V variant of thiocillin did not protect *B. cereus* from predation and *B. cereus* cells producing the T4V thiocillin variant allowed *M. xanthus* predator cells to grow. To our knowledge, this is the first report of a bacterial specialized metabolite that negatively influences *M. xanthus* viability.

Our analysis of the genomes of 109 *B. cereus* strains leads to the conclusion that a small clade of *B. cereus* acquired the thiocillin BGC, likely through a horizontal gain of function event, enabling this subset of strains to produce this specialized metabolite. PacBio sequencing revealed that *B. cereus* strain T lacks the BGC for thiocillin production while strain 14579 possesses it. Further analysis of publicly available *B. cereus* metagenomes revealed that 5 strains closely related to 14579 also possess the BGC and therefore likely can produce thiocillin whereas no other members of the broader *B. cereus* group possess genes related to thiocillin production. In support of our hypothesis, it is known that other genera, including *Streptomyces*, possess a similar BGC and are capable of thiocillin production. Because these bacteria are also soil inhabitants and live in similar ecological niches as Bacilli, horizontal gene transfer from *Streptomyces* species may account for the observed acquisition of this BGC within the Bacilli (Bleich et al., 2015).

**Figure 6:**
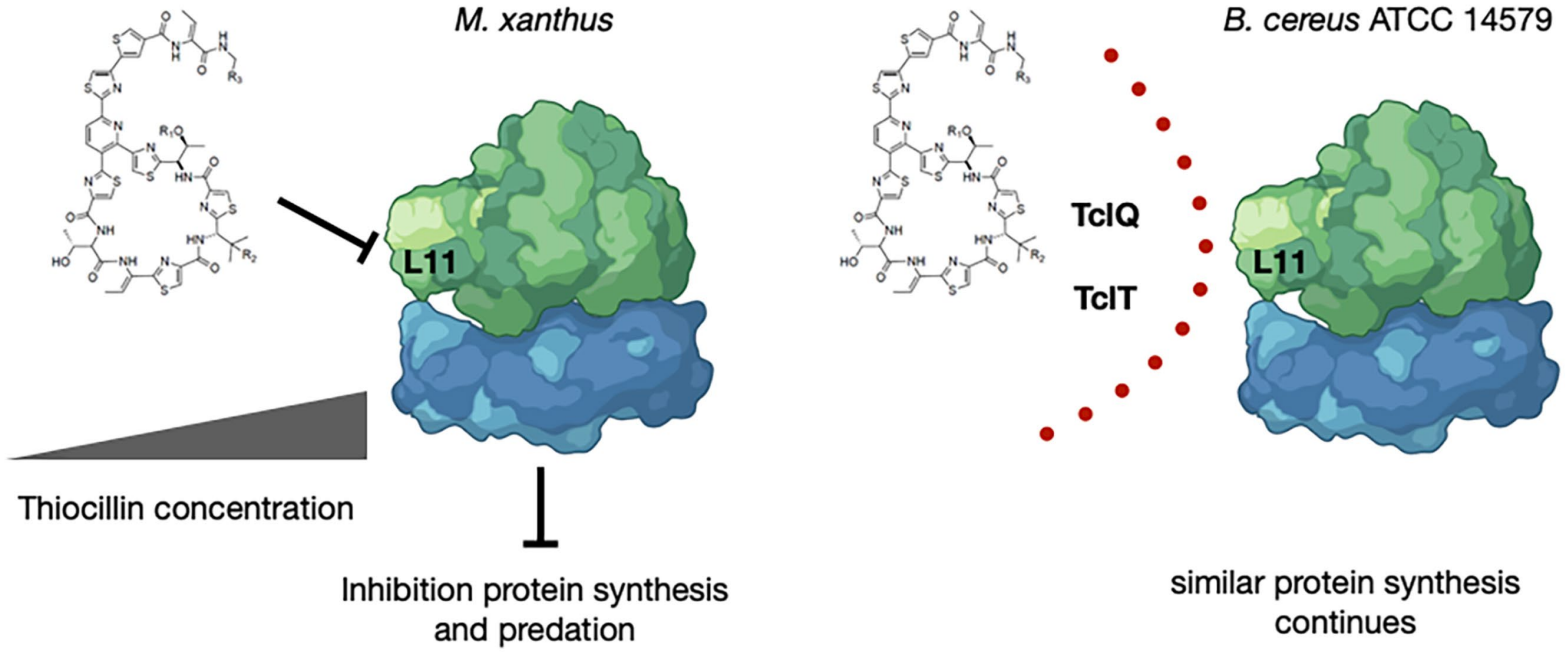
Model for Inhibition by and Protection from Thiocillin During Predation. Thiocillin is known to inhibit the L11 interaction with 23S rRNA within the 50S subunit of the ribosome to affect translation. *B. cereus* ATCC 14579, a thiocillin producer, encodes two L11 paralogs, TclQ and TclT, whereas *M. xanthus* and other *B. cereus* strains do not. TclQ and TclT most likely provide a mechanism for immunity from thiocillin within thiocillin-producing *B. cereus* strains. Thiocillin affects the fitness of *M. xanthus* and therefore provides an advantage against predation. “Created with BioRender.com.”

It is known that thiocillin acts to inhibit L11 interactions with 23S rRNA to affect translation (Cameron et al., 2002; Harms et al., 2008). Thus, the identification of two paralogs of L11 supports the proposal that gene dosage may provide a mechanism for immunity from thiocillin that would otherwise inhibit translation for thiocillin producers (Acker et al., 2009; Wieland Brown et al., 2009; Bowers et al., 2010). The paralogs of L11 encoded within the thiocillin BGCs are identical within the clade (Supplementary Figure S6) yet are distinct from the native copy of encoded by *rplK* located near *rpoA* and *rpoB*. The identity and conservation of TclQ and TclT within the thiocillin-producing clade of 5 closely related strains of *B. cereus* is consistent with recent acquisition via horizontal gene transfer. We speculate that *M. xanthus* is inhibited by thiocillin at concentrations suitable to allow for inhibition of protein translation via L11 (Figure 6). Detailed studies of thiocillin entry into *M. xanthus* cytoplasm and its mechanism of action are ongoing. Overall, this work demonstrates that thiocillin is an important contributor to survival of *B. cereus* ATCC 14579 during interspecies interactions with the predator *M. xanthus*. This specialized metabolite study extends our understanding of the chemical weaponry utilized between organisms known to interact with *M. xanthus*.

## Data availability statement

The datasets presented in this study are deposited in the NCBI database under accession numbers CP130491, CP138336, and CO138337.

## Author contributions

SM: Conceptualization, Formal analysis, Methodology, Visualization, Writing – original draft, Writing – review & editing, Investigation. OD: Methodology, Software, Visualization, Writing – review & editing. SA: Methodology, Software, Writing – review & editing. FS: Methodology, Software, Writing – review & editing. SK: Writing – review & editing. ES: Conceptualization, Supervision, Writing – review & editing. JK: Conceptualization, Funding acquisition, Project administration, Supervision, Writing – review & editing.

## Footnotes

^1^ http://rast.nmpdr.org/

## Supplementary material

SUPPLEMENTARY FIGURE S1 Thiocillin affects surface spreading of *M. xanthus* cells. Shown are enlarged pictures taken from Figure 1 (24h) to emphasize that only a few *M. xanthus* cells are visible outside of the *B. cereus* ATCC 14579 prey spot compared to sensitive prey strains *B. cereus* T and *∆tclE-H*. The prey spot location is indicated by a black ring and the distance of *M. xanthus* traveled outside of the prey spot is indicated by a double arrow line. With both sensitive strains (*B. cereus* T and *∆tclE-H*) we see consumption of the prey spot and *M. xanthus* moving beyond the initial prey spot. For the predation resistant strain ATCC 14579 we see most of the prey spot remaining and only a few *M. xanthus* cells outside of it.

Supplementary FIGURE S2 Thiocillin protects *B. cereus* ATCC 14579 from predation by *M. xanthus*. *B. cereus* strains (prey) were grown to a certain cell density (see Materials and Methods) and nutrients were removed by washing the cells multiple times. Prey cells were mixed with the predator *M. xanthus* in a ratio of 50:1, spotted on CFL Agar plates and incubated at 32°C. Pictures were taken after different times of incubation at 15x magnification. Predation is visible by cell lysis (*) whereas competition/predation resistance is indicated by minimal loss of cells (O). Fruiting body formation can be seen after 48h as an indicator that predator *M. xanthus* sensed a drop down in nutrients and is starving (black square). Strains *∆tclE-H::tclE (C9A)*, *∆tclE-H::tclE (C5A)*, *∆tclE-H::tclE (C7A)* and *∆tclE-H::tclE (C2S)* represent in frame deletions of the tclE gene that where complement with tclE point mutations leading to amino acid changes in the thiocillin molecule that disrupt the thiazolyl ring. Strain *∆tclE-H::tclE (A78)* produces a thiocillin variant with a larger thiazolyl ring. All these strains have no known antimicrobial activity against Gram-positive bacteria and do not induce matrix formation in *B. subtilis*.

Supplementary FIGURE S3 Purified Thiocillin rescues sensitive strains from predation by *M. xanthus*. The predation sensitive strains *B. cereus* ATCC 14579 *∆tclE-H::tclE* (T4V) was tested in predation assays with the predator *M. xanthus* with and without purified thiocillin (dissolved in DMSO). *B. cereus* strain *∆tclE-H::tclE* (T4V) makes a variant of the thiocillin molecule that has lost the antimicrobial function of the molecule. The strain is sensitive to predation (comparing column III to I and II). The addition of purified thiocillin protected the sensitive strain from predation (column IV). Increasing concentrations of thiocillin enhanced the predation protective effect. Pictures were taken after 24 h.

Supplementary FIGURE S4 Purified Thiocillin affects surface spreading and eating by *M. xanthus*. Shown are enlarged pictures within Figure 2 and Supplementary Figure S3. The pictures on the left show the prey strain mixed with *M. xanthus* and 330ng of purified thiocillin. The right column shows the prey strain mixed with *M. xanthus* and 990ng of purified thiocillin. The black ring indicates the prey spot and the distance traveled by *M. xanthus* outside of the prey spot is indicated by a double arrow line. At low thiocillin concentrations we see *M. xanthus* outside of the prey spots, an indicator of predation. Additionally, we see predation indicated by more lysis of the prey spot (areas marked by black square).

Supplementary FIGURE S5 Thiocillin variant T4V does not protect from predation by *M. xanthus*. The predation sensitive strains and *B. cereus* ATCC 14579 *∆tclE-H::tclE* (T4V) were tested in predation assays with the predator *M. xanthus* with and without the purified thiocillin TV4 variant molecule (dissolved in DMSO). *B. cereus* strain *∆tclE-H* does not make thiocillin and *∆tclE-H::tclE* (T4V) makes a variant of the thiocillin molecule that has lost the antimicrobial function of the molecule. All strains are sensitive to predation (comparing column III to I and II). The addition of purified thiocillin protected all sensitive strains from predation (column IV). Increasing concentrations of thiocillin enhanced the predation protective effect. Pictures were taken after 24 h.

Supplementary FIGURE S6 Alignment of L11 homologs TclT and TclQ. Protein sequence Alignment of L11 homologs from strains FSL_M8_0473, FSL_R5_0811, ATCC 14579, NCTC2599 and BDRD_Cer4 (Figure S6 A) and tctT and tclQ (Figure S6 B).
