# Supplementary figures and images for: Thiocillin contributes to the ecological fitness of Bacillus cereus ATCC 14579 during interspecies interactions with Myxococcus xanthus
Source: Front Microbiol. 2023 Nov 24;14:1295262. doi: 10.3389/fmicb.2023.1295262 (PMC10704990; doi:10.3389/fmicb.2023.1295262)

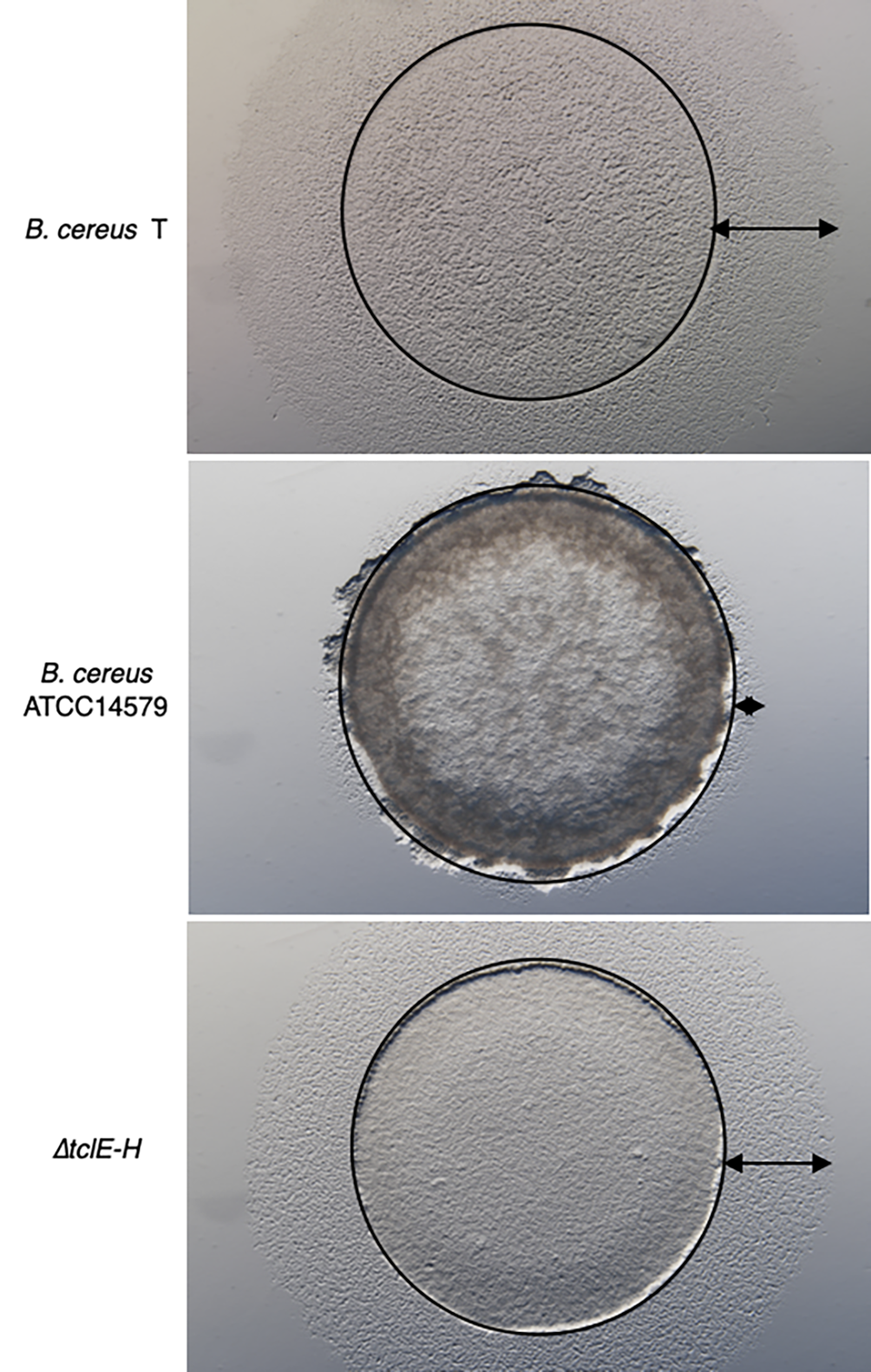

Supplement: SUPPLEMENTARY FIGURE S1 — Thiocillin affects surface spreading of M. xanthus cells. Shown are enlarged pictures taken from Figure 1 (24h) to emphasize that only a few M. xanthus cells are visible outside of the B. cereus ATCC 14579 prey spot compared to sensitive prey strains B. cereus T and ∆tclE-H. The prey spot location is indicated by a black ring and the distance of M. xanthus traveled outside of the prey spot is indicated by a double arrow line. With both sensitive strains (B. cereus T and ∆tclE-H) we see consumption of the prey spot and M. xanthus moving beyond the initial prey spot. For the predation resistant strain ATCC 14579 we see most of the prey spot remaining and only a few M. xanthus cells outside of it. [file Image_1.TIF]

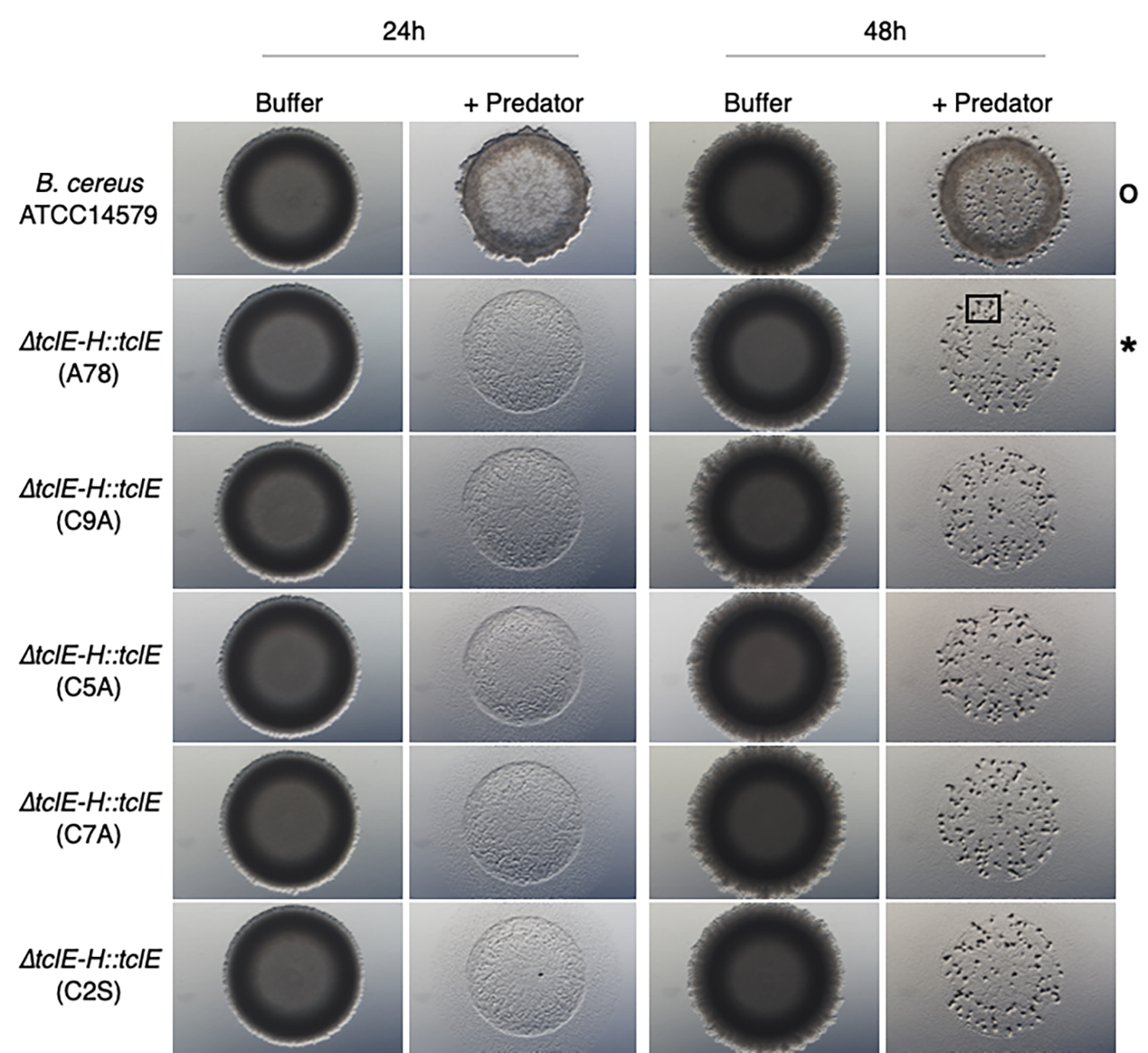

Supplement: Supplementary FIGURE S2 — Thiocillin protects B. cereus ATCC 14579 from predation by M. xanthus. B. cereus strains (prey) were grown to a certain cell density (see Materials and Methods) and nutrients were removed by washing the cells multiple times. Prey cells were mixed with the predator M. xanthus in a ratio of 50:1, spotted on CFL Agar plates and incubated at 32°C. Pictures were taken after different times of incubation at 15x magnification. Predation is visible by cell lysis (*) whereas competition/predation resistance is indicated by minimal loss of cells (O). Fruiting body formation can be seen after 48h as an indicator that predator M. xanthus sensed a drop down in nutrients and is starving (black square). Strains ∆tclE-H::tclE (C9A), ∆tclE-H::tclE (C5A), ∆tclE-H::tclE (C7A) and ∆tclE-H::tclE (C2S) represent in frame deletions of the tclE gene that where complement with tclE point mutations leading to amino acid changes in the thiocillin molecule that disrupt the thiazolyl ring. Strain ∆tclE-H::tclE (A78) produces a thiocillin variant with a larger thiazolyl ring. All these strains have no known antimicrobial activity against Gram-positive bacteria and do not induce matrix formation in B. subtilis. [file Image_2.TIF]

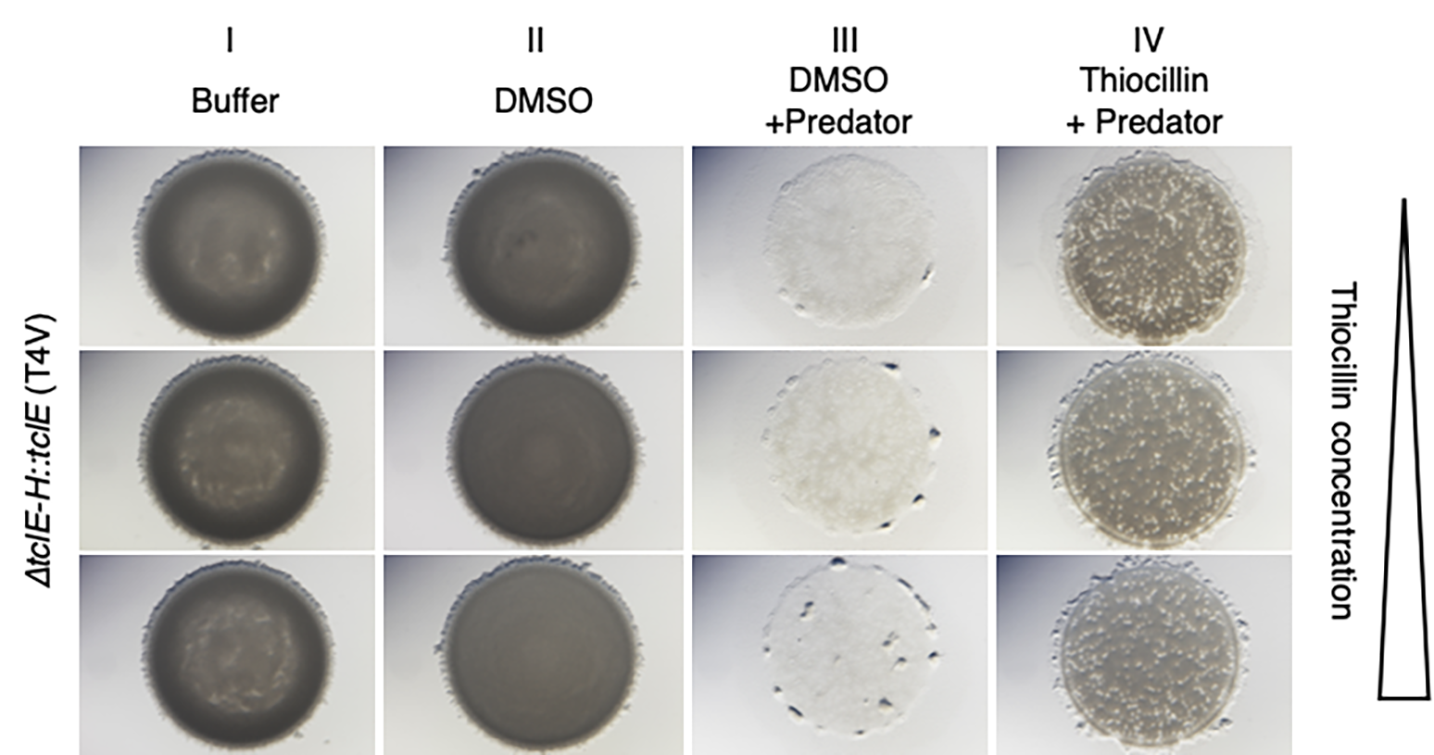

Supplement: Supplementary FIGURE S3 — Purified Thiocillin rescues sensitive strains from predation by M. xanthus. The predation sensitive strains B. cereus ATCC 14579 ∆tclE-H::tclE (T4V) was tested in predation assays with the predator M. xanthus with and without purified thiocillin (dissolved in DMSO). B. cereus strain ∆tclE-H::tclE (T4V) makes a variant of the thiocillin molecule that has lost the antimicrobial function of the molecule. The strain is sensitive to predation (comparing column III to I and II). The addition of purified thiocillin protected the sensitive strain from predation (column IV). Increasing concentrations of thiocillin enhanced the predation protective effect. Pictures were taken after 24 h. [file Image_3.TIF]

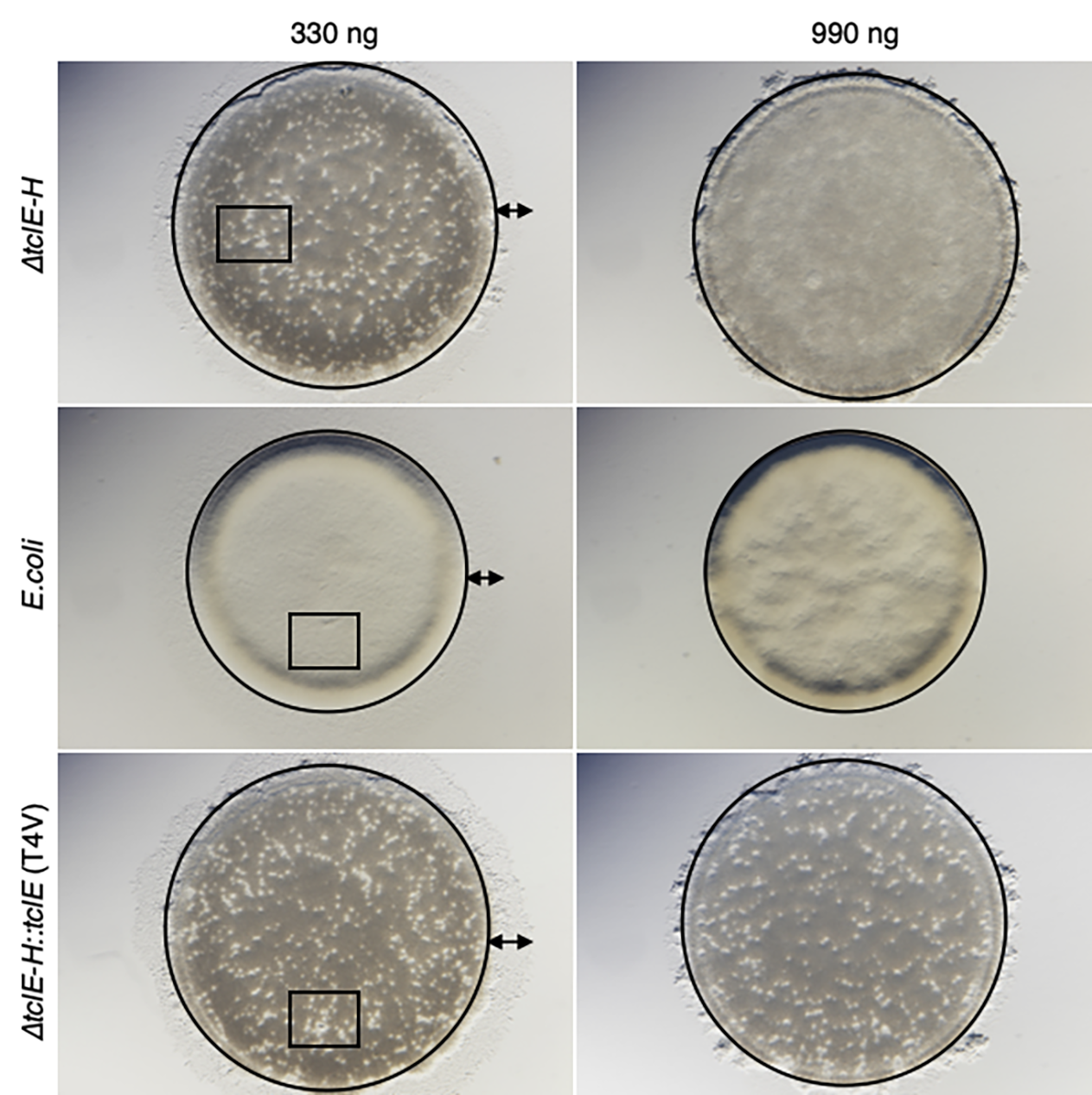

Supplement: Supplementary FIGURE S4 — Purified Thiocillin affects surface spreading and eating by M. xanthus. Shown are enlarged pictures within Figure 2 and Supplementary Figure S3. The pictures on the left show the prey strain mixed with M. xanthus and 330ng of purified thiocillin. The right column shows the prey strain mixed with M. xanthus and 990ng of purified thiocillin. The black ring indicates the prey spot and the distance traveled by M. xanthus outside of the prey spot is indicated by a double arrow line. At low thiocillin concentrations we see M. xanthus outside of the prey spots, an indicator of predation. Additionally, we see predation indicated by more lysis of the prey spot (areas marked by black square). [file Image_4.TIF]

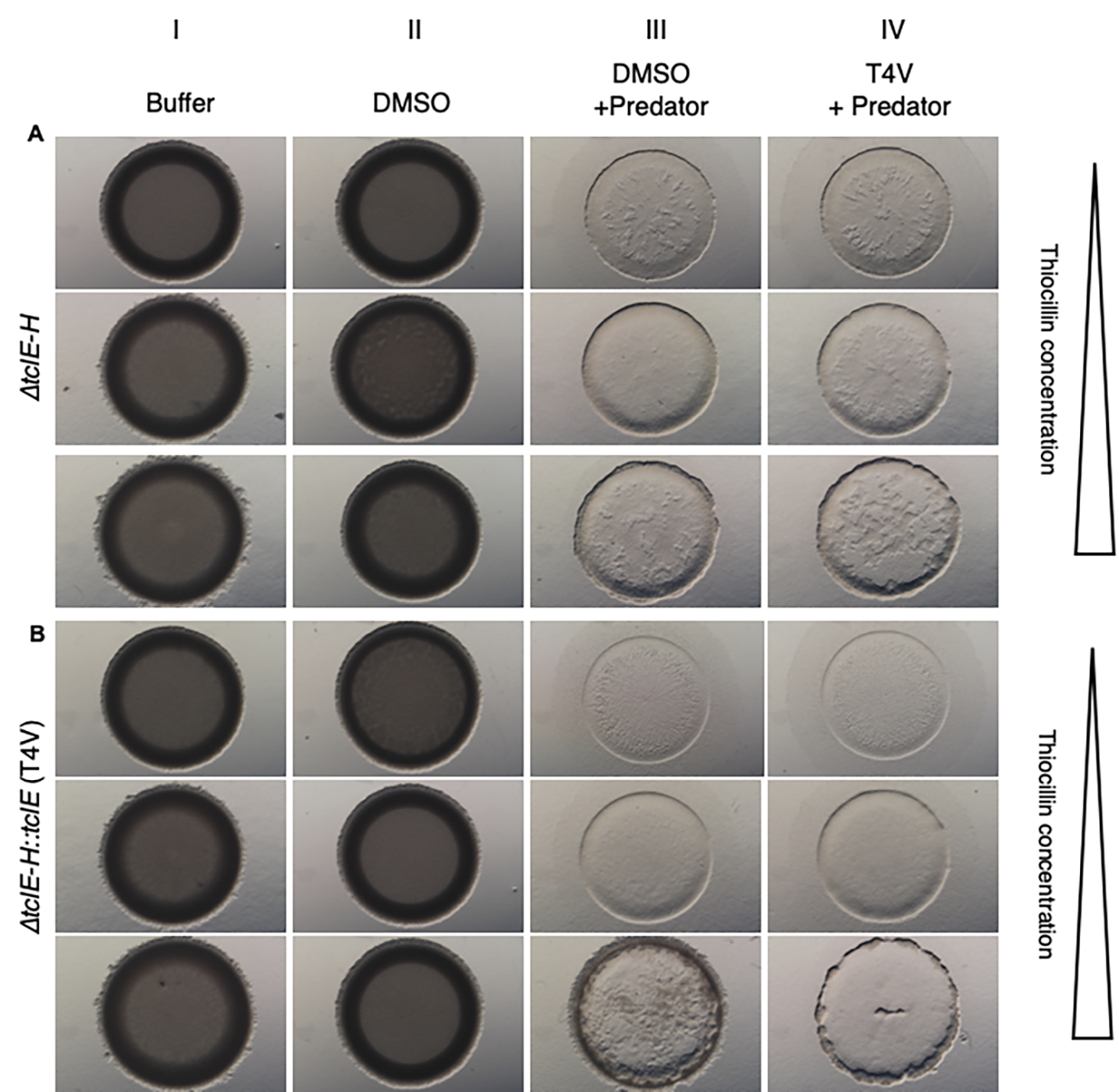

Supplement: Supplementary FIGURE S5 — Thiocillin variant T4V does not protect from predation by M. xanthus. The predation sensitive strains and B. cereus ATCC 14579 ∆tclE-H::tclE (T4V) were tested in predation assays with the predator M. xanthus with and without the purified thiocillin TV4 variant molecule (dissolved in DMSO). B. cereus strain ∆tclE-H does not make thiocillin and ∆tclE-H::tclE (T4V) makes a variant of the thiocillin molecule that has lost the antimicrobial function of the molecule. All strains are sensitive to predation (comparing column III to I and II). The addition of purified thiocillin protected all sensitive strains from predation (column IV). Increasing concentrations of thiocillin enhanced the predation protective effect. Pictures were taken after 24 h. [file Image_5.TIF]

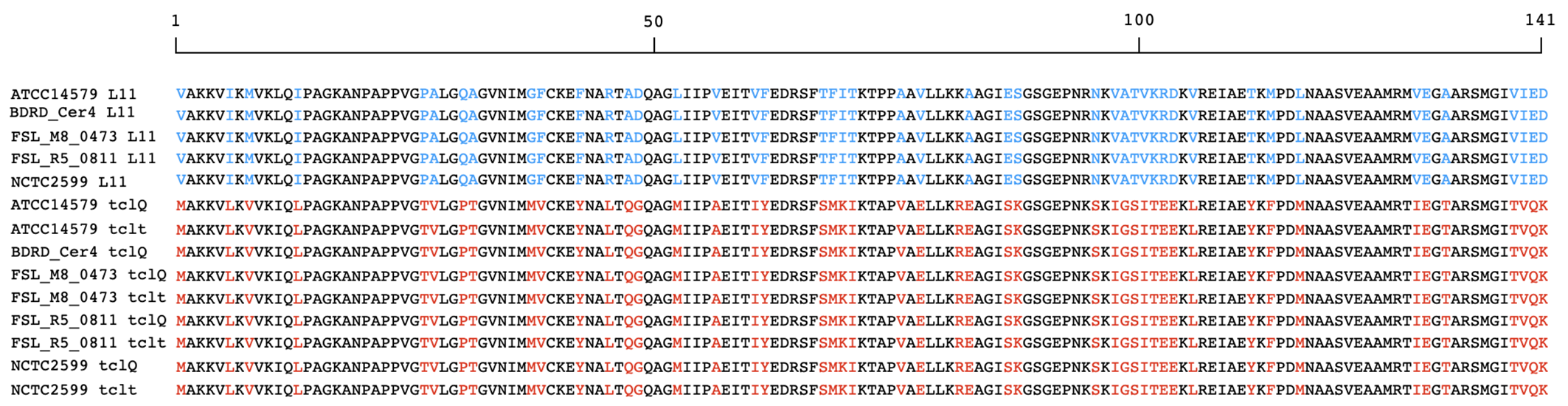

Supplement: Supplementary FIGURE S6 — Alignment of L11 homologs TclT and TclQ. Protein sequence Alignment of L11 homologs from strains FSL_M8_0473, FSL_R5_0811, ATCC 14579, NCTC2599 and BDRD_Cer4 (Figure S6 A) and tctT and tclQ (Figure S6 B). [file Image_6.TIF]
